# Deposition-Induced Thermo-Mechanical Strain Behaviour of Magnetite-Filled PLA Filament in Fused Filament Fabrication Under Varying Printing Conditions

**DOI:** 10.3390/polym17172430

**Published:** 2025-09-08

**Authors:** Boubakeur Mecheri, Sofiane Guessasma

**Affiliations:** 1 ESTP, Campus de Troyes, 1 Rue Fernand Sastre, 10430 Rosières-près-Troyes, France; bmecheri@estp.fr; 2 Research Unit BIA UR1268, INRAE, Rue Geraudiere, F-44316 Nantes, France

**Keywords:** magnetic PLA filament, thermo-mechanical behaviour, residual stress, strain evolution, functional composite filament

## Abstract

Residual stresses and internal strains in 3D printing can lead to issues such as cracking, warping, and delamination—challenges that are amplified when using functional composite materials like magnetic PLA filaments. This study investigates the thermo-mechanical strain evolution during fused filament fabrication (FFF) of magnetite-filled PLA using an integrated methodology combining strain gauge sensors, high-resolution infrared thermal imaging, and synchrotron X-ray microtomography. Printing parameters, including nozzle temperature (190–220 °C), build platform temperature (30–100 °C), printing speed (30–60 mm/s), and cooling strategy (fan on/off) were systematically varied to evaluate their influence. Results reveal steep thermal gradients along the build direction (up to −1 °C/µm), residual strain magnitudes reaching 0.1 µε, and enhanced viscoelastic creep at elevated platform temperatures. The addition of magnetic particles modifies heat distribution and strain evolution, leading to strong sensitivity to process conditions. These findings provide valuable insight into the complex thermo-mechanical interactions governing the structural integrity of magnetically functionalized PLA composites in additive manufacturing.

## 1. Introduction

Additive manufacturing (AM) has emerged as a transformative technology that has garnered significant attention over the past few decades [1]. Its capability to produce highly intricate and functional components has positioned it as a promising alternative to traditional manufacturing methods [2]. AM typically involves the layer-by-layer deposition of material based on a digital model [3]. One of its major strengths lies in the precise spatial control of material placement [4], enabling custom-designed parts with minimal need for tooling [5]. This flexibility also facilitates the development of novel and multifunctional material systems, including smart and adaptive materials [6]. Owing to its rapid fabrication capabilities, AM is widely adopted across diverse sectors such as biomedical engineering, aerospace, automotive, architecture, and product prototyping [7].

In addition, AM has been widely applied in the fabrication of electronics and sensors, where the ability to integrate functional materials and miniaturized structures is crucial. Recent studies have demonstrated the feasibility of printing flexible and high-performance devices such as energy storage and sensing systems [8]. The versatility of AM processes supports the use of a wide range of materials, with fused filament fabrication (FFF) emerging as a cost-effective method for fabricating polymer-based structures [9,10].

Among the commonly used materials in FFF, polylactic acid (PLA) and acrylonitrile butadiene styrene (ABS) have received extensive attention [11,12]. Early research by Ahn et al. [13] demonstrated how part orientation contributes to anisotropic mechanical behaviour in ABS-printed components. More recent efforts have focused on enhancing FFF through advanced composite feedstocks [14], such as ceramic-based [15] and carbon-fibre-reinforced polymers [16,17,18], aiming to offset mechanical limitations associated with porosity and process-induced defects. In this line, Caminero et al. [19] demonstrated that the inclusion of graphene nanoplatelets in PLA significantly enhances thermal conductivity, dimensional accuracy, and mechanical properties, while Wang et al. [20] reported that carbon-fibre-reinforced PLA composites achieve superior mechanical strength and thermal resistance.

Within this context, magnetic composites based on PLA have drawn interest due to their potential in applications such as electromagnetic shielding, sensor design, and actuation systems [21,22]. For example, Buj-Corral et al. [23] analyzed porosity in copper-reinforced PLA and showed that a combination of layer height, print speed, and printing temperature is needed to achieve a porosity content of 20%. Yan et al. [24] demonstrated the printability of iron oxide/PLA composites for microwave absorption, achieving optimal performance with a combination of 6 wt% graphene and 11 wt% Fe_2_O_3_. Ukir et al. [25] further explored the influence of fused filament processing parameters on mechanical and thermal performance in metal-reinforced PLA composites such as copper and bronze. Their study shows that the addition of metallic particles does not necessarily lead to improved mechanical strength, although PLA–copper composites allowed the best thermal resistance. More recently, Yan et al. [24] investigated Fe_3_O_4_/PLA composites and reported excellent microwave absorption and electromagnetic shielding properties, further confirming the multifunctionality of magnetically loaded PLA filaments.

Accurately measuring the forces and strains generated during filament deposition remains a significant technical challenge, particularly when thermally and mechanically heterogeneous composites such as magnetic PLA are used. Residual stresses and internal strains arise due to rapid thermal gradients during the repeated heating and cooling cycles of FFF [26], often resulting in dimensional distortions, interfacial stress concentrations, and potential delamination or cracking [27,28,29]. These phenomena are intensified in magnetically loaded systems due to altered thermal conductivity and viscosity. Conventional stress measurement methods often lack the resolution and adaptability needed to capture these dynamic changes, underscoring the importance of integrating innovative diagnostic techniques [30]. Recent advancements have introduced fibreoptic sensors such as fibre Bragg grating sensors (FBGSs) for in situ stress and thermal monitoring in AM processes [31,32]. PLA and other common AM materials like PET-G, ABS, and composites with carbon or aramid fibres have been successfully paired with FBGSs for embedded monitoring. Various approaches have also been developed for strain sensing using additive manufacturing. For example, Shin et al. [33] created crack-based strain gauges using FFF for biomedical monitoring, while Zeng et al. [34] developed high-temperature sensors via direct ink writing. Košir et al. [35] demonstrated how FFF process optimization can enhance both conductivity and mechanical performance in piezoelectric polyvinylidene fluoride for active piezoelectric sensing. Hashemi Sanatgar et al. [36] also reported that PLA/CNT nanocomposites can be engineered as piezoresistive strain-sensing materials, combining structural performance with real-time monitoring capability.

Strain gauge sensors have also been used to monitor thermal expansion and residual stress in metal- and polymer-based AM. Arbogast et al. [37] investigated thermal expansion in metal-based AM, observing that the behaviour of 3D-printed steel was comparable to that of conventional alloys. Chen et al. [28] employed fibre Bragg grating sensors to monitor strain and stress in FFF printed lamellae, revealing wide variation in residual stresses up to the MPa scale and a negative correlation with layer thickness.

In FFF, thermal management plays a decisive role not only in residual stress development but also in the quality of interlayer bonding and the formation of defects such as voids. Rapid substrate cooling can accelerate heat extraction, reducing residual stress accumulation; however, it may also hinder molecular diffusion between adjacent layers, thereby weakening the weld strength and promoting void formation. Conversely, elevated substrate temperatures improve chain mobility and diffusion, leading to enhanced interlayer adhesion but at the cost of increased residual stresses due to slower stress relaxation dynamics. This trade-off has been highlighted in recent investigations. For instance, Costa et al. [38] demonstrated that substrate cooling can create non-uniform temperature fields that significantly affect bonding quality and dimensional stability. Similarly, Su et al. [39] theoretically analyzed how substrate temperature and fan-assisted cooling jointly affect both stress redistribution and interlayer bonding, pointing out that rapid convective cooling promotes void formation and reduces weld strength. Experimental validation on high-temperature polymers confirms these effects. A study on PEEK showed that insufficient platform heating leads to poor bonding strength and increased porosity at the interfaces, whereas maintaining higher substrate temperatures enhances interlayer adhesion [40]. Reviews on interlayer bonding in FFF [41,42] further establish that fan-assisted cooling, while beneficial for reducing cycle time and residual stress, often compromises interlayer weld strength and mechanical performance by inducing premature solidification and limiting molecular diffusion.

In this regard, complementary IR thermography has also been used to monitor FFF, as shown by Nascimento et al. [32] and Caltanissetta et al. [43], who demonstrated that in situ infrared monitoring provides valuable insight into thermal behaviour and stress evolution in PLA composites. More recently, Shmueli et al. [44] demonstrated the power of combining infrared thermal imaging with in situ wide-angle X-ray scattering (WAXS) to simultaneously track the evolution of crystalline structures and thermal gradients during PLA extrusion. Their results revealed that nozzle orientation and local cooling history critically influence crystallinity development, interfacial diffusion, and, ultimately, the mechanical integrity of the printed part. This coupling of IR imaging with X-ray scattering represents a significant step toward multi-modal monitoring of additive manufacturing, as it links thermal dynamics with microstructural evolution at the filament interfaces

Recent advances have also addressed multifunctional PLA-based composites with enhanced mechanical and adaptive properties. Radzuan et al. [45] developed carbon-fibre-reinforced PLA filaments with significantly improved wear resistance and mechanical performance, highlighting the potential of fibre reinforcement strategies to overcome intrinsic brittleness in PLA. In parallel, Sanaka et al. [46] reported the design of heat-responsive PLA/PU/MXene nanocomposites exhibiting remarkable enhancements in tensile and flexural strength, thermal stability, and shape memory behaviour. These developments illustrate the current trend of tailoring PLA-based filaments not only for improved thermo-mechanical reliability but also for multifunctionality. In this context, our investigation of magnetically functionalized PLA filaments contributes to the growing research field on multifunctional composites by addressing their thermo-mechanical performance under realistic FFF printing conditions.

In this study, we combine thermal imaging and strain gauge sensors to monitor strain evolution under various thermal cycling and processing parameters. Complementary high-resolution techniques, including synchrotron X-ray microtomography and bolometric infrared imaging, provide microstructural insight into strain localization and thermal effects. Experiments conducted at the European Synchrotron Radiation Facility (ESRF), beamline BM05, enabled structural visualization down to 10 µm under in situ thermal cycling. This integrated approach offers novel understanding of the coupling between thermal and mechanical phenomena in magnetically loaded PLA composites, with implications for structural integrity, actuation, sensing, and electromagnetic shielding applications in additive manufacturing.

## 2. Materials and Methods

### 2.1. Materials and Processes

**Figure 1:**
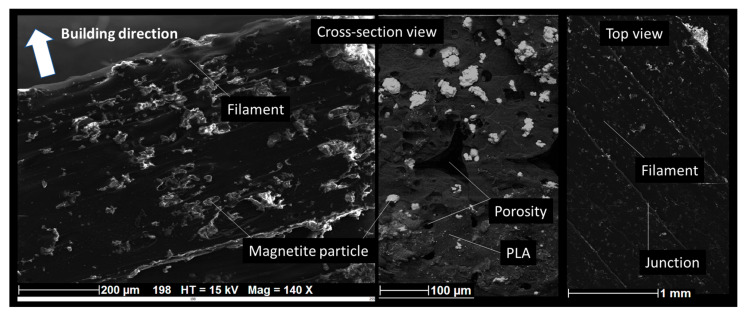
SEM micrographs illustrating the microstructure of 3D-printed PLA–magnetite composites at various magnifications.

The filament employed as feedstock material for fused filament fabrication (FFF) is a 1.75 mm diameter magnetite-filled PLA filament commercially available from Protopasta (Protoplant Inc., Vancouver, WA, USA). This composite filament consists of a PLA matrix heavily loaded with iron powder of less than 250 µm in size, giving it both magnetic responsiveness and increased density by a factor 1.5 compared to PLA and reaching a value of 1.85 g/cm^3^. The melting temperature of the filament is close to 155 °C. SEM analysis shows that the average volume content of magnetite particles is close to 13% (Figure 1). In the SEM images, magnetite particles appear as bright features due to their electrical conductivity, making elemental analysis unnecessary to distinguish them from the PLA matrix. The magnetite particle content was carried out by analyzing 2D micrographs taken perpendicular to the surface to minimize perspective bias. The images were then segmented using ImageJ software v1.53 (NIH, Bethesda, MD, USA), and the resulting counts of nearly 250 particles were converted into volume content.

Table 1 shows the specification of PLA–magnetite composite filament, including composition, physical properties, and magnetic characteristics. Among the key facts about the filament is the size and shape variability of the iron oxide particles. Such heterogeneity can influence both thermal and mechanical behaviour: particle clustering may create local variations in thermal conductivity and cooling rates, while larger particles or agglomerates can act as stress concentrators, leading to variability in load transfer and increased scatter in mechanical properties. However, the small content and the size threshold of 100 µm makes it unlikely that these particles significantly influence the bulk thermal or mechanical properties of the composite.

The additive manufacturing process used in this study is based on the fused filament fabrication (FFF) principle. The FFF equipment used is a commercial printer named SuperRacer from FLSUN (Lespinasse, France), equipped with a hardened steel nozzle to resist abrasion from the iron filler.

In this process, a thermoplastic filament—in our case, magnetite-filled PLA—is driven through a heated nozzle, where it is melted and deposited layer by layer onto a fixed build platform maintained at a controlled temperature (up to 100 °C). The nozzle supports extrusion temperatures as high as 260 °C, mounted on a gantry system, and traces the programmed toolpath while moving in the *X*–*Y* plane. The printing head is raised progressively along the *Z*-axis to accommodate subsequent layers.

**Figure 2:**
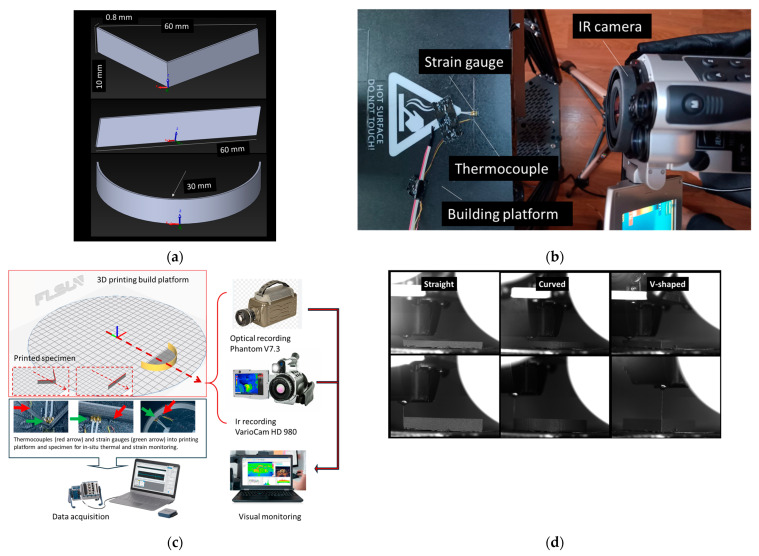
Experimental setup: (**a**) geometries of the stripline used to evaluate thermo-mechanical behaviour during the filament deposition process. (**b**) Apparatus used for monitoring thermal cycling and strain during 3D printing. (**c**) Schematic of the experimental setup showing positioning of strain gauges (green arrows), thermocouples (red arrows), and infrared camera for in situ monitoring. (**d**) Optical recording showing the building up of the three types of striplines.

The tested specimens consist of different stripline geometries with a fixed thickness. Figure 2a illustrates the CAD representations of three stripline geometries designed to assess thermo-mechanical behaviour during the fused filament fabrication (FFF) process (Figure 2c):

- Top geometry: a V-shaped angled strip with a total length of 60 mm, width of 10 mm, and thickness of 0.8 mm. This configuration introduces a geometric discontinuity to induce strain localization and simulate stress concentration during thermal cycling and deposition.
- Middle geometry: a straight stripline with identical length (60 mm) and cross-sectional profile, serving as a baseline geometry to evaluate strain and temperature evolution under uniform printing conditions, free of intentional curvature or angular features.
- Bottom geometry: a curved strip with a 30 mm radius of curvature and a 60 mm arc length. This geometry is used to investigate the influence of non-linear deposition paths on residual stress development and thermal gradient distribution, simulating common shapes encountered in functional parts.

The stripline geometries were selected because they serve as the fundamental elements for most prints that combine curved, edge, and straight lines. A former study by the authors showed rapid thermal cycling in linear striplines [47]. In the present study, the selected geometries represent typical case studies where printability can vary significantly due to factors such as stepper motor acceleration and deceleration, uneven heat generation and accumulation, and fluctuations in material flow caused by changes in printing speed during these phases.

Each configuration allows localized monitoring of strain and thermal fields during printing, aiding in the understanding of deformation mechanisms influenced by shape, deposition direction, and thermal loading. The experimental setup is illustrated in Figure 2b, where the fixed printing parameters include a 0.4 mm nozzle diameter, a displacement speed of 150 mm/s, and a layer height of 0.2 mm. No bottom or top layers are used, and neither support structures nor adhesion rafts are applied. The main variables investigated are the printing speed, cooling strategy, extrusion, and base temperatures, with settings summarized in Table 2.

All slicing operations are carried out using Cura 4.4 by Ultimaker company (Utrecht, The Netherlands). These parameters are chosen to tune interlayer bonding and residual stresses, accounting for the higher viscosity and increased thermal inertia associated with the magnetite-filled PLA composite.

For thermal tracking, K-type micro-thermocouples (Omega Engineering company, Stamford, CT, USA); 50 µm in size and supporting a temperature range between −200 and +1350 °C) were positioned adjacent to the strain gauges and at the PLA–bed interface. Temperature and strain were acquired simultaneously using NI hardware (NI 9213, NI 9237, NI cDAQ-9174).

A VarioCam HD 980 infrared camera (InfraTec GmbH, Dresden, Germany) provided complementary thermal imaging (1207 × 850 pixels, <30 mK sensitivity, 7.5–14 µm range, with a frame rate of 30 Hz). The camera is calibrated in the range 0 °C and 500 °C, and autofocus is disabled. The pixel size is set to 65 µm. Both measurement setups are interfaced with an NI cDAQ-9174 chassis, which collects and synchronizes experimental data through a dedicated acquisition system.

In our experimental setup (Figure 2), strain gauges and thermocouples were directly bonded to the build platform along the toolpath centreline, enabling synchronized monitoring of thermal and mechanical responses during filament deposition. The combination of thermal input (nozzle and bed temperatures), extrusion rate, and cooling conditions governs interlayer bonding and residual stress evolution in the printed parts.

### 2.2. Mechanical and Strain Analysis

The strain measurement procedure during FFF is schematically illustrated in Figure 2b. Strain gauges were bonded to the printed striplines, while thermocouples and an infrared camera simultaneously recorded the thermal field. All sensors were connected to a National Instruments acquisition system to synchronize strain and temperature data in real time.

To gain a detailed understanding of the strain distribution during the thermal cycling of magnetic PLA filament deposition, we employed wire resistance strain gauges capable of both static and dynamic strain monitoring during the fused filament fabrication (FFF) process. Sensor selection was guided by several criteria, including high sensitivity to microstrain, non-invasiveness, and compatibility with the constrained environment of 3D printing. The strain gauge used in this study is the KFH-1.5-120-C1-11L3M3R model from Omega Engineering (Norwalk, CT, USA). It has a nominal grid resistance of 120 Ω with a tolerance of ±0.35% and a gauge factor (GF) of 1.98 ± 1.5%. The transverse sensitivity is 0.3%. Its coefficient of thermal expansion is α = 10.8 × 10^−6^/K. The temperature coefficient of the gauge factor is specified as 93 ± 10 × 10^−6^/K within the operating temperature range of −10 °C to +45 °C. The thickness of the constantan sensing element is 3.8 µm, while the polyimide backing has a thickness of 45 ± 10 µm. It is constructed from constantan alloy (55% copper, 45% nickel), which is sensitive to both mechanical and thermal strain. The active sensing grid is 1.5 mm long and supported by a polyimide film. The strain gauges are bonded directly onto the printed specimens using cyanoacrylate fast-acting (10 to 15 s) glue allowing a hold up of 180 kg/cm^2^ and that withstands temperatures as high as 150 °C.

The mechanical deformations along the axis of the gauge are obtained from the proportional changes in resistance ($∆R_{S}$) by a piezoresistive effect. The total measured strain $\left(\varepsilon_{G}\right)$ reads:

(1) $$\varepsilon_{G}=\left(∆R_{S}/R_{n}\right)/G_{F}$$

where $G_{F}$ is the gauge factor and represents the ratio between the relative change in nominal resistance $R_{n}$ and the relative change in length or strain.

As the gauge is thermally sensitive, the total strain can be regarded as a linear sum of the real strain $\varepsilon_{R}$ independent of the temperature effect, on the one hand, and the thermal strain $\varepsilon_{T}$:

(2) $$\varepsilon_{G}={\varepsilon_{T}+\varepsilon }_{R}$$

Prior to the tests, the strain gauges were calibrated according to the manufacturer’s specifications. A polynomial correction was applied to account for thermal sensitivity, ensuring that the extracted strain values represent mechanical deformation independent of temperature effects.

The apparent strain caused by temperature changes ($\varepsilon_{T}$) is evaluated using a third-order polynomial correction provided by the manufacturer:

(3) $$\varepsilon_{m}\;\left(µm/m\right)=31.18+2.629\times T\;\left({}^{\circ}C\right)-5.78\times 10^{-2}\times T^{2}\;\left({}^{\circ}C\right)+2.41\times 10^{-4}\times T^{3}\;\left({}^{\circ}C\right)$$

where T is the measured temperature in °C.

Strain evolution is tracked in response to key printing parameters such as nozzle speed, filament flow rate, and extrusion and bed temperatures.

## 3. Results and Discussion

### 3.1. Heating Analysis

**Figure 3:**
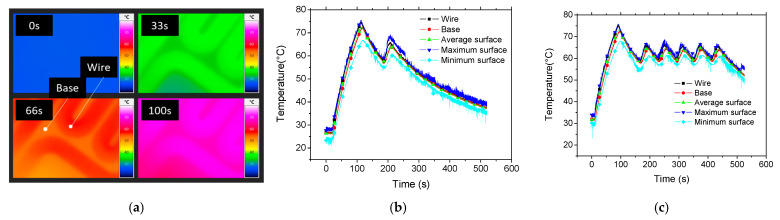
Analysis of the heating stage using a high-resolution infrared camera. (**a**) Image sequence showing the building platform as it approaches the target temperature of 60 °C. (**b**) Temperature evolution at a specific location on the building platform considering only one cycle before cooling, (**c**) similar temperature evolution for multiple temperature regulation steps along with the average temperature over the entire sensed area.

In order to examine any deviation between the prescribed base temperature and the achieved one on the printing platform, the heating of the building platform is monitored. Figure 3 shows a typical sequence for a target base temperature of 60 °C.

Figure 3a demonstrates that the heating is not uniform within the building platform, where the highest temperatures are recorded within the heating wires compared to the surrounding base. Over time, as shown in Figure 3a, the heat gradually spreads across the platform, reducing the temperature gradient. However, even at 100 s, residual non-uniformities persist, indicating localized temperature variations due to the geometry and layout of the embedded heating elements.

Figure 3b,c complement this observation by presenting the PID-controlled temperature evolution over time at various locations on the platform, as identified in Figure 3a. All curves show a rapid initial temperature rise followed by a peak and a subsequent gradual decline, which is consistent with the heating and regulation behaviour of the system. The measurements confirm that areas closer to the heating wires (e.g., wire) reach temperatures as high as 72 °C before oscillating between 57 °C and 64 °C. The maximum recorded temperature on the building platform even reaches 76 °C. The decrease in the temperature at the cooling stage occurs with a low rate of 0.08 °C/s. Within this stage, the difference between the minimum and maximum temperatures can be as high as 6 °C. This supports the conclusion that thermal gradients persist during the heating phase, potentially influencing the thermal history of printed material and thus the resulting mechanical properties. Figure 3c illustrates the temperature evolution at different locations on the building platform under closed-loop control using a PID-based heating system. After the initial ramp-up phase—where all curves show a steep increase in temperature—the system overshoots the setpoint (around 60 °C), which is a typical response when the proportional gain is relatively high and the integral term continues to accumulate during the rise. Following the peak, the controller adjusts by reducing power, and the temperature begins to drop toward the setpoint. What is particularly noticeable is the presence of oscillations after the initial peak with an amplitude of 5 °C. These oscillations, which persist through the steady-state phase, indicate a typical underdamped PID response. This is characteristic of a system where the derivative term is not strong enough to fully dampen the oscillations, or the tuning prioritizes a fast response at the expense of some stability. The oscillation amplitudes vary slightly across different points on the platform, suggesting that the local thermal inertia and proximity to the heating elements influence how each location responds to the same global control input. Despite these oscillations, the temperature remains within a relatively narrow band around the target, which suggests that the PID controller is functioning effectively overall but could benefit from further tuning to minimize residual oscillations and improve thermal uniformity. This thermal cycling may have implications for material deposition uniformity and residual stresses in the printed parts.

**Figure 4:**
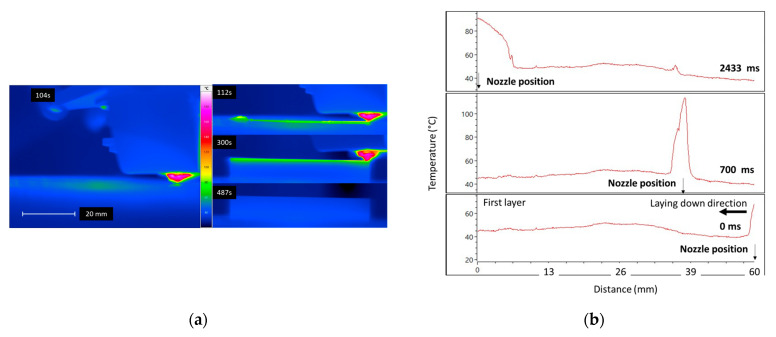

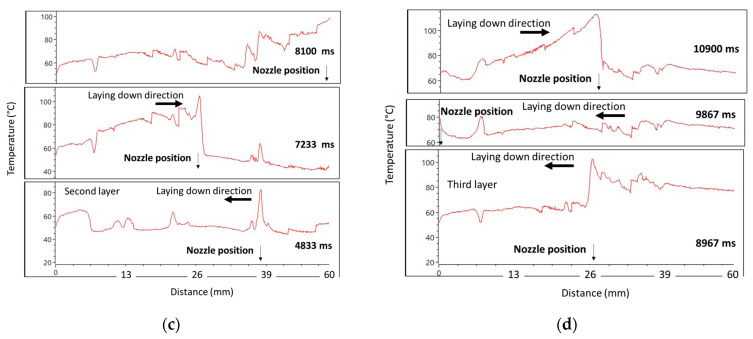
Thermal cycling and strain levels monitored during the laying down process for the reference condition. (**a**) IR snapshots at different printing stages, Temperature profile along filament laying down in the building platform’s (**b**) first, (**c**) second, and (**d**) third layer.

Figure 4a captures a typical filament deposition process monitored with an IR camera under reference conditions, with the nozzle and base temperatures set to 220 °C and 60 °C, respectively. To avoid the noise coming from the build platform edge reflections, the latter is covered with black tape, which is a common strategy when using IR cameras to reduce reflective artifacts. The timestamps (e.g., 104 s, 112 s, 300 s, and 487 s) correspond to critical stages of printing, including nozzle positioning before deposition, first-layer application, final-layer deposition, and the post-cooling phase. The thermal images reveal the evolution of heat distribution and dissipation throughout the process. The filament appears to be deposited continuously, as evidenced by uniform layering in the initial and final stages. Cooling exhibits anisotropic behaviour, with faster heat dissipation observed along the build direction compared to the direction of deposition.

Figure 4b–d present a sequence of thermal images captured by the IR camera, showing the temperature evolution during the initial stages of filament deposition on the build platform. Figure 4b specifically captures the first 2 s of nozzle movement from right to left along the stripline, corresponding to the deposition of the first layer. During this period, the temperature rises from approximately 62 °C at the edge to a peak of 107 °C after 700 ms. The snapshot at 2433 ms marks the completion of the first layer. A distinct temperature peak near the 32 mm position indicates a significant disruption in the filament. The nozzle’s displacement rate is approximately 30 mm/s between 0 and 700 ms and slightly lower, at around 25 mm/s, between 0 and 2433 ms due to deceleration at the endpoints.

Figure 4c illustrates the temperature evolution during the formation of the second layer. At 4833 ms, the temperature profile appears jagged, likely due to discontinuous material deposition caused by the increased height of the strain gauge and thermocouple, which may have altered the material flow at the contact points. As the nozzle reverses direction to begin the second segment of the stripline, a sharp yet uneven temperature drop is observed in the trailing region, with a gradient reaching up to −2 °C/mm (as seen in the snapshot at 7233 ms). By the end of the second layer at 8100 ms, the temperature profile remains discontinuous, influenced by surface roughness and irregular material deposition.

Figure 4d illustrates the temperature evolution along the stripline during the deposition of the third layer at three different time instances. In the top graph (10,900 ms), as the nozzle moves from left to right, a pronounced temperature peak exceeding 100 °C is observed just before the nozzle position, followed by a steep drop, indicating active filament deposition and rapid cooling. The middle graph (9867 ms) corresponds to the return pass of the nozzle during the second layer deposition, where a lower and more irregular temperature profile is recorded. The jagged nature of the curve suggests disrupted material flow, likely caused by the elevated surface profile around the strain gauge and thermocouple, which can alter nozzle–substrate interaction. In the bottom graph (8967 ms), marking the mid-position from the third layer deposition, a clear temperature peak around 100 °C appears near the nozzle position, but the temperature profile before and after is uneven and discontinuous, likely due to variations in surface roughness and material continuity from prior layers. Collectively, these profiles reflect the complex thermal dynamics influenced by deposition direction, sensor-induced disturbances, and substrate conditions.

**Figure 5:**
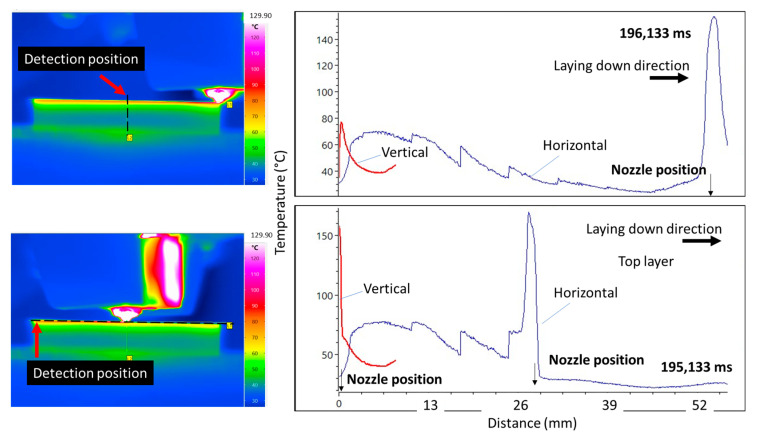
Vertical and horizontal temperature profiles along the stripline at the top layer during the deposition process, shown for the reference condition and two different printing durations. The detection position is indicated by red arrows for both vertical and horizontal profiles.

Figure 5 presents vertical and horizontal temperature profiles along the stripline at the top layer for two different printing times: 195,133 ms and 196,133 ms. The horizontal profiles (blue curves) reflect the surface temperature distribution along the deposition path, while the vertical profiles (red curves) capture the thermal gradient across the stripline height at the centre position. In both cases, the horizontal profiles exhibit distinct temperature peaks at the nozzle position, with temperatures reaching above 140 °C in the 196,133 ms case and exceeding 160 °C at 195,133 ms, indicating active material deposition. The temperature gradually decreases behind the nozzle due to cooling.

The vertical profiles show sharp, narrow peaks directly beneath the nozzle, signifying strong thermal concentration during extrusion. Notably, the vertical temperature peaks are steeper and reach higher values in the earlier time step (195,133 ms), suggesting increased heat accumulation possibly due to repeated layer deposition or localized heating. These results highlight the thermal asymmetry and localized heating effects during the filament laying process, where the largest thermal gradient is witnessed along the building direction, with a typical magnitude of −1 °C/µm, and a lower one (−0.1 °C/µm) along the filament deposition path.

**Figure 6:**
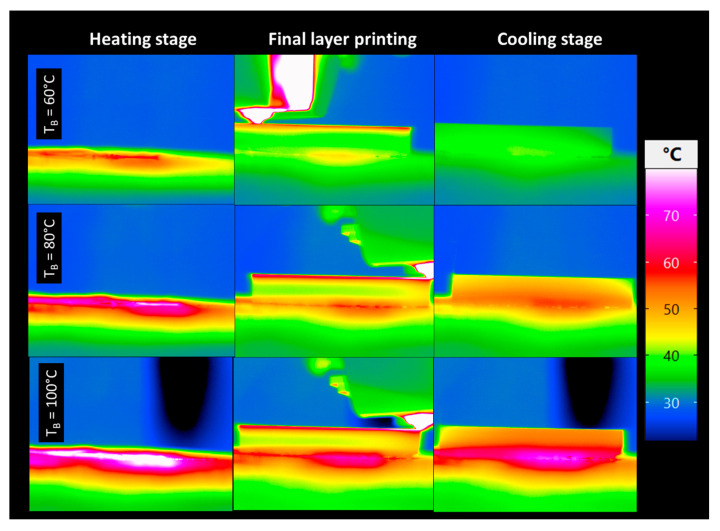
Effect of the base temperature on the laying down process captured using IR camera.

Figure 6 presents infrared thermographic images captured during three stages of the printing process—heating, final layer deposition, and cooling—for three different build platform temperatures (TB = 60 °C, 80 °C, and 100 °C). In the heating stage, a clear heat accumulation at the centre is observed due to the presence of the strain gauge that dissipates 53 mW and the thermocouple, with higher base temperatures (Tbs) leading to non-uniform and elevated preheating of the substrate at the sensor locations. During the final layer printing, a sharp thermal contrast is evident at the nozzle position, where localized heating peaks above 70 °C, especially at higher platform temperatures, indicating intensified thermal interaction and enhanced potential for interlayer bonding. Notably, at Tb = 100 °C, the heat penetration into the lower layers is deeper and more homogeneous, as indicated by the expanded high-temperature zone (red-pink region). In the cooling stage, samples printed at higher Tb retain more thermal energy, resulting in a slower and more uniform cooling profile. This suggests that elevated platform temperatures improve thermal continuity across layers, which may enhance fusion quality and reduce residual stresses in the printed part.

Overall, these results underline that the platform heating stage introduces inherent thermal heterogeneity, which directly influences the subsequent thermal cycling of deposited layers. Similar observations were made by Costa et al. [38], who demonstrated through numerical modelling that non-uniform platform heating generates local gradients that propagate into the deposited material. Cattenone et al. [48] also confirmed via finite element analysis that spatial variations in initial substrate temperature contribute to localized distortions during FFF. In addition, Casavola et al. [49] experimentally highlighted that residual stresses correlate strongly with substrate thermal gradients rather than with uniform base heating. Taken together, these studies support our conclusion that the heating stage plays a decisive role in defining the initial thermal history, which governs subsequent stress development during the printing process.

Beyond these global trends, the thermal stress analysis must also account for local influencing factors. Variations in material flow at the contact points of strain gauges and thermocouples may disturb deposition continuity, creating localized perturbations in heat transfer and stress fields. Furthermore, the heating heterogeneity of the build platform, evidenced in Figure 3 and Figure 6, generates additional temperature gradients that amplify residual stress formation. These localized effects are particularly relevant for interlayer bonding: excessive substrate cooling can promote void formation at the interface, while uneven heating may induce premature solidification or poor polymer diffusion between layers [32,43]. As a result, the measured thermal stresses reflect both the intrinsic process dynamics and extrinsic perturbations related to the experimental configuration. Considering these aspects is essential to assess the net effect of substrate temperature and cooling strategy on mechanical performance and printing quality.

### 3.2. Effect of Base Temperature

**Figure 7:**
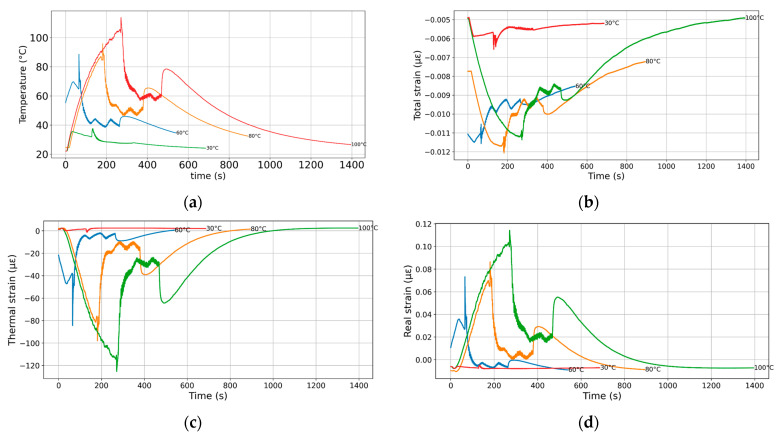
Influence of base temperature on the evolution of mechanical and thermal strain during the deposition process. (**a**) Temperature profiles recorded by the embedded thermocouple within the build platform. (**b**) total measured, (**c**) thermal, and (**d**) real strain evolution as a function of the printing time.

In order to assess the mechanical and thermal strain evolution as affected by the base temperature, Figure 7 illustrates temperature versus time profiles for four samples initially heated to different peak temperatures (30 °C, 60 °C, 80 °C, and 100 °C). The building platform temperature is assessed from the thermocouple embedded in the building platform close to the position of the strain gauge. All curves show an initial rapid rise in temperature, followed by a peak, after which a cooling phase ensues. The red curve, representing the sample heated to 100 °C, exhibits the highest peak, followed by a complex cooling profile characterized by noticeable fluctuations before gradually declining. This peak corresponds to the moment when the nozzle first reaches the thermocouple position. The subsequent fluctuating decay phase reflects the deposition of successive layers, during which the heat source moves progressively farther from the initial layer. These fluctuations arise from the base temperature regulation, following the same scheme illustrated in Figure 3. For a printing base temperature of 100 °C, this fluctuating decay phase extends from approximately 300 to 500 s. A slight temperature increase then occurs, marking the end of the deposition process. Finally, a smooth and steady cooling phase continues until around 1400 s.

For base temperatures of 80 °C, 60 °C, and 30 °C, the temperature curves show progressively lower peaks and shorter durations of thermal activity. Each curve reaches its peak when the nozzle first contacts the thermocouple, after which a cooling phase begins. At 80 °C and 60 °C, this phase includes pronounced fluctuations due to active base temperature regulation during layer deposition, extending roughly to 500 and 450 s, respectively. These are followed by a slight temperature rise indicating the end of deposition, then a smooth decline. In contrast, the 30 °C curve shows a much lower peak and minimal fluctuations, with a rapid transition into a steady cooling phase, reflecting reduced thermal input and regulation at the lower base temperature.

Figure 7b shows the corresponding total strain evolution as a function of the printing time for the same printing conditions. The measured strain profiles reveal a clear dependence on the base temperature, with higher temperatures inducing greater initial contraction followed by more pronounced recovery. At 100 °C and 80 °C, the strain drops significantly during the early phase—corresponding to the thermal peak—then gradually increases as the material cools and internal stresses relax. The 60 °C curve shows a similar trend but with notable oscillations during the deposition phase, reflecting thermal instabilities and less efficient stress release. In contrast, the 30 °C sample exhibits only a slight initial strain change and remains largely stable throughout, indicating minimal thermal deformation and limited stress evolution due to the reduced heat input.

Figure 7c illustrates the evolution of the thermal strain component as a function of time for various base temperatures, revealing clear distinctions in thermo-mechanical response. At higher base temperatures (100 °C and 80 °C), the thermal strain undergoes a pronounced initial contraction, reaching minimum values of approximately −125 µε and –100 µε, respectively, before gradually recovering over time. This behaviour reflects the significant thermal expansion followed by contraction as the material cools and the temperature gradient decreases. The curves for 60 °C and 30 °C show progressively smaller strain amplitudes, with 60 °C displaying moderate fluctuations and a peak contraction around −65 µε, while the 30 °C case remains nearly flat, indicating negligible thermal deformation due to minimal heating. The timing of the minima aligns with the end of the deposition phase, highlighting the dominant role of base temperature in driving thermally induced strain during the process.

Figure 7d illustrates the real strain, independent of thermal expansion, which increases markedly with base temperature, indicating a strong base temperature dependence of the material’s time-dependent mechanical response. At lower temperatures (30 °C and 60 °C), the real strain remains low and decays rapidly, suggesting predominantly elastic behaviour with minimal viscoelastic or structural relaxation. In contrast, at higher temperatures (80 °C and 100 °C), the real strain reaches significantly higher peaks and relaxes much more slowly over time, consistent with enhanced viscoelastic creep or stress redistribution processes that are thermally and mechanically activated.

These findings are consistent with those of Chen et al. [28], who observed through FBG-based monitoring that residual stresses relax progressively as layers accumulate. Similarly, Alzyod and Ficzere [50] demonstrated numerically that platform temperature has only a minor effect compared to nozzle temperature or layer thickness, while Casavola et al. [49] confirmed experimentally that stress evolution is primarily governed by deposition strategy and thermal gradients. This supports our conclusion that thermal history, rather than bed heating alone, is the decisive factor controlling stress redistribution in FFF.

### 3.3. Effect of Forced Cooling

**Figure 8:**
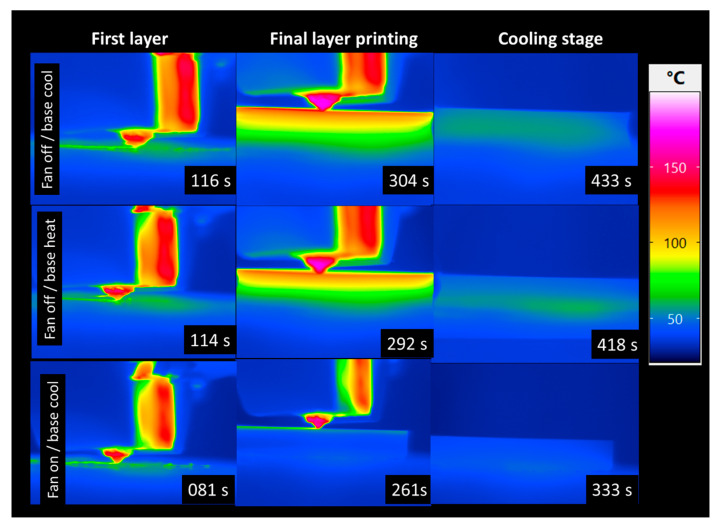
Effect of forced cooling on the laying down process captured using IR camera.

Figure 8 illustrates the effects of forced cooling during the laying down process, combining infrared (IR) camera measurements of surface temperature with strain gauge data capturing mechanical strain evolution.

The IR images reveal how forced cooling alters the temperature distribution and accelerates cooling rates across the printed part. In this figure, three heating and cooling modes during fused filament fabrication are compared: fan off with base cooling after printing, fan off with continuous base heating after printing, and fan on with base cooling at the end of the print. In the fan off/base cooling condition, higher residual temperatures persist throughout printing and into the cooling stage, indicating slower, more uniform cooling driven only by natural convection. The fan off/base heating mode further elevates the temperature across the build surface, resulting in thermal gradient as shown in the snapshot at 418 s. In contrast, the fan on/base cooling mode results in significantly lower surface temperatures in all stages, with much faster and more localized cooling evident via the reduced thermal footprint in the final layer and cooling stage. This demonstrates that forced convection dramatically accelerates cooling rates and steepens thermal gradients, which can reduce cycle time but may increase residual stress and deformation risk.

**Figure 9:**
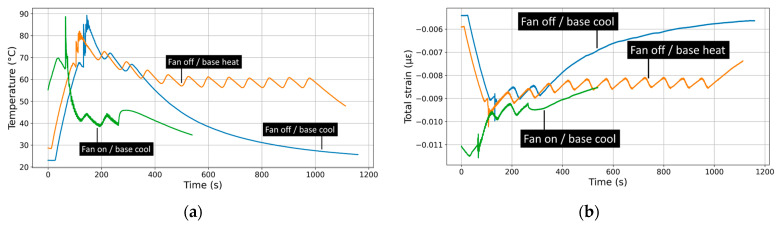

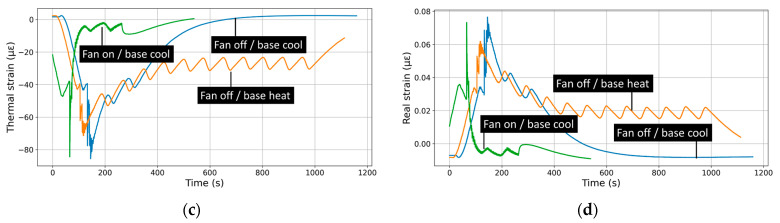
Influence of forced cooling on the evolution of mechanical and thermal strain during the deposition process. (**a**) Temperature profiles recorded by the embedded thermocouple within the build platform. (**b**) Total measured, (**c**) thermal, and (**d**) real strain evolution as a function of the printing time.

Figure 9a illustrates the effect of forced cooling on the first-layer temperature evolution during printing, measured by a thermocouple. Under the fan on/base cooling condition, the temperature rises quickly up to 90 °C, marking the beginning of the filament deposition, but reaches a lower peak and begins dropping rapidly almost immediately during the laying down process, indicating that forced convection effectively extracts heat and limits temperature buildup at the first layer. The smooth temperature decrease indicates no PID regulation effect. In contrast, the fan off/base heating condition shows higher temperature levels and a steady-state regime with no gradual cooling profile, suggesting that without forced airflow, heat dissipates only by conduction and natural convection, leading to sustained elevated temperatures. The temperature fluctuations around 60 °C in the steady-state regime results from the PID regulation. The fan off/base cooling condition further increases the peak close to 90 °C and maintains the highest overall temperature during the laying down process up to 400 s, before reaching a more gradual and smooth cooling regime and a faster cooling compared to the fan off/base heating condition.

Figure 9b presents the total measured strain at the first layer under different cooling conditions over time. In the “fan off/base cooling” condition, a negative strain build-up prior to printing occurs during the first 100 s. During the laying down process, a contraction is maintained up to 300 s, corresponding to the duration of the printing process. The magnitude of the total strain decreases more sharply as time progresses, indicating a limited buildup of strain due to the base cooling. This suggests that while the base is cooling, the material experiences an overall contraction over time, leading to an increase in strain.

In the “fan off/base heating” condition, the negative strain is maintained for a longer time, stabilizing between 200 and 1000 s and slightly oscillating between −0.008 and −0.009 µε. This behaviour suggests that the combination of cooling and base heating maintains a constant oscillatory contraction during the laying down due to the PID control allowing for a strain accumulation. Finally, in the “fan on/base cooling” condition, the magnitude of the negative strain increases up to 0.011 µε, allowing larger contraction to take place.

Figure 9c displays the thermal strain component over time for the same three cooling conditions. This plot isolates the strain component due to temperature effects, helping distinguish thermal expansion/contraction behaviour from mechanical deformation. Under “fan off/base heating”, thermal strain becomes highly negative (reaching values near −70 με). During the printing process (>100 s), thermal contraction occurs as the structure heats up unevenly. This suggests a large temperature gradient prior to printing or delayed expansion due to the printing process, which allows a thermal equilibrium to be reached. Over time, the strain eventually recovers toward zero if the base temperature decreases (>1000 s), likely due to heat saturation or reduced thermal gradient between the base and the layered structure. In the “fan off/base cooling” condition, thermal strain drops rapidly, which leaves no residual contraction after 600 s. During the printing process (from 180 s up to 400 s), the contraction decreases, likely due to more heat accumulation in the upper layers. The “fan on/base cooling” case shows the least thermal strain (closer to 0 με) and a smoother curve. This demonstrates that active airflow with a cooled base effectively controls temperature gradients, minimizing thermal contraction and fluctuations, thereby maintaining thermal stability in the first layer.

Figure 9d illustrates the evolution of real strain—mechanical strain independent of thermal effects—under three different cooling conditions: “fan on/base cooling” (green), “fan off/base cooling” (orange), and “fan off/base heating” (blue). The results reveal a clear influence of cooling strategy on mechanical deformation. In the “fan off/base heating” scenario, real strain peaks around 0.06 με and decays slowly over time, indicating significant mechanical distortion likely due to thermal stress accumulation without adequate cooling. In contrast, the “fan off/base cooling” case shows a similarly high initial peak but maintains elevated strain levels over time with visible oscillations, suggesting persistent and cyclic mechanical stress due to ineffective heat removal from the upper layers. The most stable behaviour is seen in the “fan on/base cooling” condition, where the real strain remains minimal and decays rapidly, reflecting effective mechanical stress relief.

Overall, these results highlight that cooling strategy significantly alters both the magnitude and relaxation of strain during FFF. Fan-assisted cooling accelerates heat dissipation and reduces thermal inertia, leading to faster strain contraction and stabilization, whereas natural convection or continuous base heating promote slower, more uniform cooling and delayed stress release. Similar effects were observed by Conceição et al. [51], who used infrared thermography to monitor FFF in real time and confirmed that cooling conditions strongly affect local temperature gradients and heat transfer dynamics. Glaskova-Kuzmina et al. [52] further showed that post-printing cooling conditions play a decisive role in mechanical performance, with rapid ambient cooling reducing tensile strength by 10–15% compared to controlled in-printer cooling. Finally, Alzyod and Ficzere [50] demonstrated numerically that although cooling rate modifies thermal history, its impact on residual stress is less pronounced than nozzle temperature or print orientation. Together, these findings reinforce our conclusion that cooling strategy primarily affects stress redistribution dynamics: forced convection enables faster stress relief but may increase residual stress sensitivity and reduce interlayer bonding, while natural convection promotes smoother but slower relaxation.

### 3.4. Effect of Print Design

**Figure 10:**
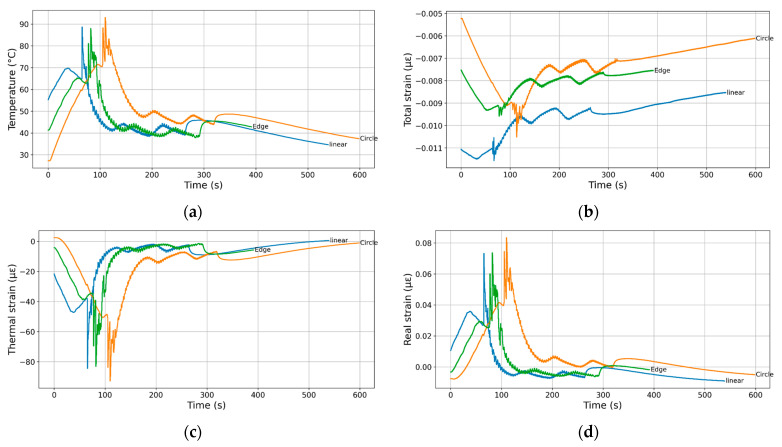
Influence of the stripline form on the thermal cycling during the deposition process. (**a**) Temperature profiles recorded by the embedded thermocouple within the build platform. (**b**) Total measured, (**c**) thermal, and (**d**) real strain evolution as a function of the printing time.

Figure 10 shows the effect of print path design on thermal cycling during the printing process. The temperature profiles at the base of the 3D printer for the three different printed geometries—circle, linear, and edge—demonstrate distinct thermal behaviours over time. Initially, all geometries exhibit a similar rise in temperature, but they diverge significantly as printing progresses (Figure 10a). The circle geometry (orange curve) reaches the highest peak temperature, exceeding 90 °C, and maintains elevated temperatures for a longer duration, indicating more sustained thermal accumulation likely due to the continuous movement and overlap of extruded paths. Both the linear and edge geometries (blue and green curves) show more fluctuation and a lower peak temperature, suggesting quicker cooling intervals between passes and less concentrated heat. These observations are consistent with the findings of Samy et al. [53], who numerically demonstrated that concentric raster patterns promote more homogeneous thermal accumulation than linear or zigzag paths, thereby reducing thermal gradients responsible for warpage.

The total strain measured at the base of the 3D printer also varies notably across the three printed geometries, reflecting the influence of thermal gradients and mechanical constraints during the printing process (Figure 10b). The circle geometry (orange curve) experiences the least compressive strain (more positive), particularly after 200 s, indicating a more uniform thermal expansion and potentially reduced mechanical resistance during cooling. In contrast, the linear geometry (blue curve) exhibits the highest compressive strain throughout the printing duration, suggesting that its directional path induces greater thermal contraction and stress accumulation. The edge geometry (green curve) shows intermediate behaviour, with strain levels between those of circle and linear, displaying a distinct fluctuation phase between 100 and 300 s that may correspond to changes in thermal input or localized mechanical constraints.

The thermal strain behaviour observed during the 3D printing process differs significantly across the three geometries (Figure 10c), revealing the impact of temperature-induced expansion and contraction. The circle geometry (orange curve) experiences the most pronounced thermal contraction, with the largest negative peak in thermal strain (below −80 µε), especially around 120 s, suggesting strong and localized thermal gradients due to the continuous circular deposition. Both linear and edge geometries, while also showing notable thermal contraction, maintain a higher (less negative) thermal strain level throughout most of the process, implying more gradual thermal variations along straight paths and a smoother recovery toward zero strain in the later stages.

Figure 10d shows real strain at the base of the 3D printer over time for the same three geometries. The “circle” geometry (orange line) exhibits the highest peak strain, reaching around 0.08 µε, with pronounced oscillations during laying down that decay more slowly compared to the others. This suggests greater residual stresses and thermal gradients due to its continuous curved path. The “linear” and “edge” geometries show intermediate behaviour, with lower and shorter-lived peaks, indicating more uniform cooling during the laying down and reduced thermal stresses. Furthermore, Huang et al. [54] experimentally confirmed that the orientation of raster patterns (0°, 90°, ±45°) directly dictates the anisotropic distribution of residual stresses, supporting our findings by showing that continuous trajectories (circle) store more residual stresses, whereas linear paths promote faster relaxation.

The strong influence of nozzle temperature on strain evolution is consistent with previous reports. Chen et al. [28] highlighted that thermal parameters such as layer number and thickness govern heat accumulation and residual stress relaxation. Similarly, Alzyod and Ficzere [50] demonstrated numerically that increasing extrusion temperature amplifies residual stresses due to intensified expansion–contraction cycles. In contrast, Cerbe et al. [55] observed experimentally in PLA 4D printing that lowering nozzle temperature increases programming stresses and residual strains, while higher temperatures promote relaxation and reduce stored stresses. These apparently divergent results confirm that nozzle temperature not only drives the magnitude of transient strain but also determines whether stresses are stored or relaxed depending on the material system and thermal history. Furthermore, Glaskova-Kuzmina et al. [52] showed that post-printing cooling conditions significantly alter the mechanical performance of high-temperature polymers such as ULTEM, with rapid ambient cooling reducing tensile strength by more than 10% compared to controlled in-printer cooling. This finding supports our interpretation that nozzle temperature cannot be considered in isolation: its effect on residual stress is coupled with subsequent cooling dynamics. Taken together, these results reinforce our conclusion that nozzle temperature is the dominant parameter governing strain evolution and residual stress development, but its impact is strongly modulated by thermal history and cooling strategy.

### 3.5. Effect of Printing Temperature

**Figure 11:**
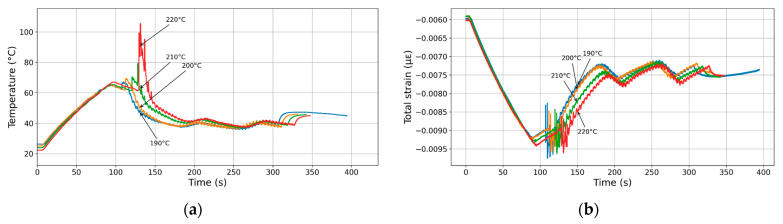

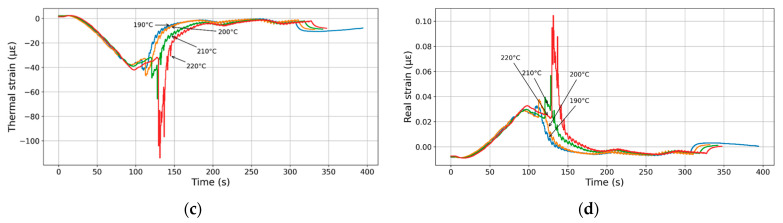
Influence of the printing temperature on the thermal cycling during the deposition process. (**a**) Temperature profiles recorded by the embedded thermocouple within the build platform. (**b**) Total measured, (**c**) thermal, and (**d**) real strain evolution as a function of the printing time.

Figure 11 exhibits the influence of printing temperature on thermal cycling. Figure 11a displays the temperature evolution over time for samples subjected to different printing temperatures: 190 °C, 200 °C, 210 °C, and 220 °C. Initially, all samples follow a similar heating profile, gradually increasing in temperature until they reach their respective peak values at around 130–140 s. At this point, a sharp drop in temperature is observed, which becomes more pronounced with increasing peak temperatures. The 220 °C curve exhibits the most significant and abrupt temperature drop, suggesting rapid heat loss due to the large difference between filament and base temperatures. During the laying down process, the temperature differences between the printing conditions diminish as the process progresses, though they still reflect the ranking based on their respective peak temperatures (around 200 s). Beyond this point, all curves converge toward a similar cooling profile, indicating that thermal equilibrium is eventually re-established, independent of the initial peak temperature.

Figure 11b presents the evolution of total strain over time for samples processed for the same printing temperatures. During the heating stage, all curves show a consistent increase in compressive strain, which reaches its maximum magnitude around 110–130 s. This is followed by a reduction in construction, with noticeable differences between the curves corresponding to each temperature. The strain recovery appears to be more delayed and less complete at high temperatures, particularly for the 220 °C condition, which exhibits the lowest strain recovery. Despite these variations, the strain profiles gradually realign, and by the end of the printing process (after approximately 180 s), all curves tend toward a common strain level. This convergence suggests that the material undergoes a similar final structural response regardless of the peak temperature, although the transient behaviour and magnitude of strain recovery are clearly influenced by the thermal exposure.

Figure 11c illustrates the evolution of thermal strain over time for the different printing temperatures. Initially, all conditions exhibit a comparable trend of increasing compressive thermal strain during the base heating stage, reaching a maximum compressive strain between 100 and 140 s. This is followed by a sharp recovery phase during the laying down process up to 200 s, where the magnitude and rate are strongly influenced by the peak temperature. Notably, the sample exposed to 220 °C undergoes the most pronounced and abrupt thermal strain drop, followed by a rapid rebound. As the process continues, differences in thermal strain between the conditions begin to narrow, although higher temperatures remain associated with more extreme transient behaviour. Eventually, beyond roughly 250–300 s, at the cooling stage, all curves begin to align, indicating that thermal strains stabilize and converge toward a common equilibrium state. This behaviour suggests that while higher processing temperatures intensify the thermal response initially, the system ultimately returns to a similar structural condition over time.

Figure 11d illustrates the evolution of real strain over time under different temperature conditions ranging from 190 °C to 220 °C. Initially, all curves display a gradual increase in strain up to around 100 s, followed by a more rapid rise that peaks between 120 and 150 s, depending on the temperature. Higher temperatures, such as 220 °C and 210 °C, result in higher peak strain values, indicating greater deformation under thermal loading. Notably, the 220 °C curve exhibits the most pronounced peak and fluctuation, reaching a strain close to 0.1 µε, while the 190 °C condition shows the lowest maximum strain and a more gradual decline after the peak. After the peak strain, all curves demonstrate a marked drop and eventual stabilization, suggesting relaxation or recovery processes. This behaviour underscores the strong influence of temperature on the material’s thermal expansion and mechanical response over time.

These findings are consistent with those of Chen et al. [28], who observed that stress evolution is strongly influenced by thermal parameters such as layer number and thickness, which govern heat accumulation. Similarly, Alzyod and Ficzere [50] found that extrusion temperature significantly increases residual stress in ABS due to larger expansion–contraction cycles. Cerbe et al. [55] demonstrated that in PLA 4D structures, lowering nozzle temperature increases programming stresses and residual strains, while higher nozzle temperatures promote relaxation. Together, these studies confirm our conclusion that nozzle temperature is the dominant factor governing transient strain behaviour and residual stress in magnetite-filled PLA composites.

### 3.6. Effect of Printing Speed

**Figure 12:**
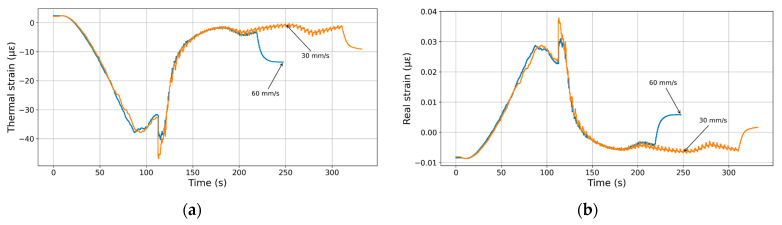
Influence of the printing speed on the thermal cycling during the deposition process. (**a**) Thermal and (**b**) real strain evolution as a function of the printing time.

Figure 12 shows the effect of printing speed on the strain evolution during the laying down process. Two main levels are selected, namely 30 mm/s and 60 mm/s. The use of a higher speed results in a short laying down process of about 100 s. Figure 12a shows the effect of the printing speed on the thermal strain evolution. Both curves initially exhibit a steep increase in thermal compressive strain, reaching a magnitude of around –45 µε at approximately 100 s, indicating significant contraction during the base heating stage. During the laying down process, the strain recovers sharply and stabilizes near –2 µε, irrespective of the printing speed. The cooling stage for both conditions occurs at two different epochs, which marks the increase in thermal strain at about 200 s and 310 s. This stage is also marked by a moderate oscillation due to the PID regulation. Overall, the data indicate that the printing speed does not greatly affect thermal strain evolution.

Figure 12b shows the same trends for the real strain where both printing conditions exhibit no marked differences. Both curves start with a steady increase in strain, peaking sharply around 120 s and with a maximum near 0.035 µε, indicating significant deformation during heating. After this peak, both curves drop quickly and stabilize at low or slightly negative strain values. Beyond 200 s, the behaviour diverges due to the fact that the cooling stage is reached at different times. Overall, the data highlight no strong influence of printing speed on strain evolution during and after thermal processing.

These observations suggest that within the investigated range, printing speed exerts only a marginal influence compared to thermal parameters. This finding is consistent with the numerical work of Alzyod and Ficzere [50], who reported that higher speeds generally reduce residual stress due to faster cooling and more uniform heat distribution, although they also noted that excessive speeds compromise dimensional accuracy and surface quality. Similarly, Cerbe et al. [55] demonstrated in PLA 4D printing that higher printing speeds increase programming forces but do not necessarily lead to higher residual strain, as cooling dynamics compensate for the added stress. In addition, Sood et al. [10] highlighted that the combined effect of print speed and orientation primarily influences dimensional distortions, while its effect on residual stresses remains secondary. Together, these studies support our conclusion that in magnetite-filled PLA, strain evolution is far more sensitive to nozzle and bed temperatures than to the printing speeds tested.

## 4. Conclusions

This study investigated the thermo-mechanical strain evolution of magnetite-filled PLA during fused filament fabrication (FFF) under varying processing conditions. Based on strain gauge monitoring, infrared thermography, and synchrotron imaging, the main findings can be summarized as follows:

- Influence of base temperature: elevated build platform temperatures (80–100 °C) promote higher strain magnitudes and slower recovery, consistent with thermally activated viscoelastic relaxation and stress redistribution. Lower temperatures (30–60 °C) result in limited strain evolution and predominantly elastic behaviour.
- Effect of cooling strategy and print path: forced convection accelerates heat dissipation but enhances residual stress accumulation, while circular deposition paths promote greater heat retention and higher strain levels compared to linear or edge paths.
- Role of nozzle temperature: higher extrusion temperatures (210–220 °C) intensify transient strain responses and delay recovery, confirming that thermal gradients and thermal history govern the development of residual stresses.
- Sensitivity to print speed: within the investigated range (30–60 mm/s), print speed shows only a marginal effect on strain evolution compared to thermal parameters, highlighting that nozzle and bed temperatures are the dominant factors controlling thermo-mechanical behaviour.

These insights underline the importance of thermal management in minimizing residual stresses in FFF and provide a framework for optimizing process parameters when printing magnetically functionalized PLA composites.

## Figures and Tables

**Table 1:** Key material properties and composition details of the magnetite-filled PLA filament.

Parameter	Value
Volume content	13% *
Additive	Ferromagnetic metal powder
Particle size	10 to 90 µm
Particle aspect ratio	0.54 to 1.00
Min bend diameter	35 mm
Melt point	155 °C
Induction at magnetic saturation	0.15 T

* based on SEM.

**Table 2:** 3D printing conditions. Reference condition in bold characters.

Parameter	Value
Pattern	**Linear**, curved, V-shaped
Printing temperature (°C)	190, 200, 210, **220**
Base temperature (°C)	30, **60**, 80, 100
Cooling strategy	Fan off, **on**
Printing speed (mm/s)	**30**, 60

## Data Availability

The original contributions presented in this study are included in the article. Further inquiries can be directed to the corresponding author.
